# Watchful waiting as a strategy to reduce low-value spinal imaging: study protocol for a randomized trial

**DOI:** 10.1186/s13063-021-05106-x

**Published:** 2021-02-27

**Authors:** Joshua J. Fenton, Anthony Jerant, Peter Franks, Melissa Gosdin, Ilona Fridman, Camille Cipri, Gary Weinberg, Andrew Hudnut, Daniel J. Tancredi

**Affiliations:** 1 Department of Family and Community Medicine, University of California, Davis, Davis, USA; 2 Center for Healthcare Policy and Research, University of California, Davis, Davis, USA; 3 Margolis Center for Health Policy, Duke University, Durham, NC USA; 4 Sutter Institute for Medical Research, Sacramento, CA USA; 5 Department of Pediatrics, University of California, Davis, Davis, USA

**Keywords:** Back pain, Diagnostic testing, Patient-doctor communication, Primary care, Overuse, X-rays/roentgenography, Computed tomography, Magnetic resonance imaging, Randomized controlled trial

## Abstract

**Background:**

Patients with acute low back pain frequently request diagnostic imaging, and clinicians feel pressure to acquiesce to such requests to sustain patient trust and satisfaction. Spinal imaging in patients with acute low back pain poses risks from diagnostic evaluation of false-positive findings, patient labeling and anxiety, and unnecessary treatment (including spinal surgery). Watchful waiting advice has been an effective strategy to reduce some low-value treatments, and some evidence suggests a watchful waiting approach would be acceptable to many patients requesting diagnostic tests.

**Methods:**

We will use key informant interviews of clinicians and focus groups with primary care patients to refine a theory-informed standardized patient-based intervention designed to teach clinicians how to advise watchful waiting when patients request low-value spinal imaging for low back pain. We will test the effectiveness of the intervention in a randomized clinical trial. We will recruit 8–10 primary care and urgent care clinics (~ 55 clinicians) in Sacramento, CA; clinicians will be randomized 1:1 to intervention and control groups. Over a 3- to 6-month period, clinicians in the intervention group will receive 3 visits with standardized patient instructors (SPIs) portraying patients with acute back pain; SPIs will instruct clinicians in a three-step model emphasizing establishing trust, empathic communication, and negotiation of a watchful waiting approach. Control physicians will receive no intervention. The primary outcome is the post-intervention rate of spinal imaging among actual patients with acute back pain seen by the clinicians adjusted for rate of imaging during a baseline period. Secondary outcomes are use of targeted communication techniques during a follow-up visit with an SP, clinician self-reported use of watchful waiting with actual low back pain patients, post-intervention rates of diagnostic imaging for other musculoskeletal pain syndromes (to test for generalization of intervention effects beyond back pain), and patient trust and satisfaction with physicians.

**Discussion:**

This trial will determine whether standardized patient instructors can help clinicians develop skill in negotiating a watchful waiting approach with patients with acute low back pain, thereby reducing rates of low-value spinal imaging. The trial will also examine the possibility that intervention effects generalize to other diagnostic tests.

**Trial registration:**

ClinicalTrials.govNCT 04255199. Registered on January 20, 2020

## Administrative information

The order of the items has been modified to group similar items (see http://www.equator-network.org/reporting-guidelines/spirit-2013-statement-defining-standard-protocol-items-for-clinical-trials/).

**Table:** 

Title {1}	Watchful Waiting as a Strategy to Reduce Low-Value Spinal Imaging: Study Protocol for a Randomized Trial
Trial registration {2a and 2b}.	NCT 04255199, ClinicalTrials.gov (January 16, 2020)
Protocol version {3}	September 9, 2020
Funding {4}	Agency for Healthcare Quality and Research (R18HS026415)
Author details {5a}	Departments of Family & Community Medicine, Pediatrics, and the Center for Healthcare Policy and Research, University of California, Davis. Sutter Institute for Medical Research, Sacramento, CA.
Name and contact information for the trial sponsor {5b}	Denise Burgess Program Official Center for Quality Improvement and Patient Safety Agency for Healthcare Research and Quality 5600 Fishers Lane Rockville, MD 20857 tel: 301-427-1318 email: denise.burgess@ahrq.hhs.gov
Role of sponsor {5c}	The study sponsor had no role in the study design; data collection, management, and analysis, and interpretation; in the writing of the report; or the decision to submit the report for publication.

## Introduction

### Background and rationale {6a}

Overutilization is increasingly viewed within the framework of patient safety [1–4]. In primary care, patients with acute low back pain frequently request diagnostic imaging, and primary care and urgent care clinicians feel pressure to acquiesce to such requests to sustain patient trust and satisfaction [5]. Spinal imaging in patients with acute low back pain poses risks from diagnostic evaluation of false-positive findings, patient labeling and anxiety [6], unnecessary treatment (including spinal surgery) with potential downstream complications [7], and added costs. The National Committee for Quality Assurance endorses the inappropriate use of spinal imaging in acute back pain as one of few valid measures of overutilization in primary care [8]. While the Choosing Wisely movement has increased physician awareness, it has not reduced the use of early spinal imaging for acute low back pain [9]. Effective approaches to reducing the use of low-value spinal imaging in primary care are needed.

Watchful waiting advice has been found an effective strategy to reduce low-value treatment (e.g., pediatric ear infections), but its role in reducing low-value testing has received minimal attention. In a Dutch randomized controlled trial, a watchful waiting strategy was acceptable to primary care patients with unexplained symptoms and reduced diagnostic blood testing [10]. In an observational analysis of data from audio-recorded primary care office visits, we found that primary care physicians who advised watchful waiting when patients requested low-value testing were 40% less likely to order the requested test than those who did not use this approach (*p* < .001), and advice to pursue watchful waiting accounted for substantial variance in low-value test ordering (*R*^2^ = 53%) [11]. Meanwhile, psychological theory suggests that physician messages could be tailored to magnify patient acceptance of a watchful waiting strategy [12, 13].

The goals of this trial are to develop a novel educational intervention designed to boost primary care physician skill in delivering a watchful waiting message to patients presenting with acute low back pain, delivered by standardized patient instructors (SPIs), and to test the effectiveness of the SPI-delivered intervention in reducing rates of low-value spinal imaging.

## Objectives {7}

Our study has three specific objectives:

Objective 1: To use key informant interviews of front-line primary care and urgent care clinicians and focus groups with primary care patients to develop and to refine a theory-informed standardized patient (SP)-based intervention designed to teach practicing clinicians how to recommend and negotiate a watchful waiting strategy when patients request low-value spinal imaging for low back pain.

Objective 2: To test in a randomized clinical trial (RCT) the effectiveness of a standardized patient instructor (SPI)-delivered clinician training in increasing the use of watchful waiting among patients with acute low back pain.

We hypothesize that the intervention will (a) reduce rates of lumbar spinal imaging among actual patients with acute back pain seen by clinicians post-intervention (adjusting for pre-RCT rates), (b) increase clinician advice to pursue watchful waiting during a follow-up visit with a regular (non-instructor) standardized patient (SP), (c) increase clinician self-reported use and efficacy of advising watchful waiting with actual low back pain patients, and (d) have no adverse impact on actual patient trust and satisfaction with physicians.

Objective 3: To assess whether intervention effects generalize to other diagnostic tests.

We hypothesize that the SP intervention will (a) decrease rates of neck imaging among actual patients with neck pain seen by study clinicians during the follow-up period (adjusting for pre-RCT rates), (b) decrease rates of overall diagnostic testing (i.e., all diagnostic imaging and blood testing) among adult patients seen by study clinicians during the follow-up period, and (c) increase clinician self-reported use of advising watchful waiting rather than imaging for patients with neck pain and other musculoskeletal pain.

## Trial design {8}

This will be a parallel group randomized trial with primary care clinicians randomized to intervention and control arms. We considered cluster randomization at the clinic level which would reduce risk that intervention clinicians will discuss their experiences with the watchful waiting messages with clinicians randomized to the control arm in the same clinic, potentially introducing contamination of that arm via their social influence on peers. However, recruitment challenges in the context of the COVID-19 pandemic made it difficult to recruit entire clinic staffs to the study simultaneously, which made a clinic-level cluster randomization scheme infeasible. We will therefore randomize clinicians overall 1:1 to intervention and control groups, which allow rolling enrollment and initiation of trial procedures in the context of ongoing pandemic mitigation. The trial is designed as a superiority trial, testing whether the intervention is superior to the control condition in reducing rates of low-value spinal imaging.

## Methods: participants, interventions, and outcomes

### Study setting {9}

We will test the simulated SPI intervention trial among clinicians practicing within 8–10 primary care or urgent care clinics affiliated with two large health systems in Sacramento, CA (USA): the UC Davis Health System and Sutter Health. Together, the two health systems operate over two dozen primary care community-based clinics in the Sacramento metropolitan area, comprising two of the four major health systems serving Sacramento with an estimated market share of 40%.

We will recruit a total of 55 primary care or urgent care clinicians for RCT enrollment. We anticipate enrolling two urgent care sites within the Sutter system where many patients with acute back pain are evaluated; no urgent care sites exist within the University of California, Davis (UCD) system, where we will only enroll primary care clinics.

### Eligibility criteria {10}

For enrollment purposes, “clinicians” are defined as primary care physicians, urgent care physicians, or nurse practitioner/physician assistants who take primary management responsibility for test ordering during routine clinical care. Clinicians will be eligible if they intend to practice at least 50% of a full-time equivalent (FTE) in adult primary care or urgent care in one of the target clinics for the trial duration and have been in active in at least 50% FTE practice within the health system for at least 2 years prior to enrollment (to enable collection of baseline testing rates). We will not include clinicians practicing < 50% FTE because of the need to accrue sufficient numbers of actual back pain patients during pre- and post-intervention phases for well-powered analyses of the primary outcome of lumbar spinal imaging ordering (see the “Sample size {14}” section). To achieve the desired sample size, we will seek to enroll clinicians from 8 to 10 primary care or urgent care clinics.

To help achieve objective 1 (developing and refining the intervention content), we have conducted key informant interviews with seven front-line clinicians. In the interviews, we elicited clinician feedback about the challenges posed in avoiding low-value imaging in patients with acute low back pain and the suitability of the study intervention in promoting effective clinician-patient communication regarding low-value spinal imaging. For key informant interviews, we recruited clinicians from within and outside the UCD Health System whom we believed would provide constructive insights. We anticipate completing at least two additional key informant interviews with clinicians. Clinicians who provided key informant interviews will not be eligible to enroll in the RCT.

We have also completed 6 patient focus groups to help achieve objective 1. For patient focus groups, we recruited English-speaking patients aged 18–65 years who had seen a primary care clinician for acute low back pain in the past 2 years. Patients were ineligible if they had chronic, persistent back pain or a history of spinal surgery. Patients were recruited using Craigslist and the UC Davis Health StudyPages website [14]. Due to the COVID-19 pandemic, we completed all focus groups online using the Zoom platform with a median of 5 patient attendees per group (*n* = 30 patients). The goal of these focus groups was to elicit patient feedback regarding the barriers and facilitators to accepting physician advice to pursue a watchful waiting strategy regarding lumbar spinal imaging. We also elicited feedback about how patients might react to specific watchful waiting messages in the context of low back pain. We plan no additional focus groups as we achieved data saturation with the completed focus groups.

### Who will take informed consent? {26a}

Research staff obtained or will obtain informed verbal assent from all potential participants prior to enrolment, including clinician enrollees for the RCT, clinician key informants for telephone interviews, and patients enrolled in focus groups. Research staff will offer enrolment to potential clinician enrollees during staff meetings. Potentially interested clinicians will receive the IRB-approved consent form via email and will provide verbal assent by either replying to the email affirmatively or by calling our research coordinator. The IRB approved omission of obtaining signatures on consent forms. Likewise, we emailed consent forms to clinician key informant and patients participating in focus groups, and these participants provided verbal assent prior to the beginning of interviews or focus groups, respectively.

### Additional consent provisions for collection and use of participant data and biological specimens {26b}

Not applicable as this trial does not involve collecting biological specimens for storage.

## Interventions

### Explanation for the choice of comparators {6b}

We will compare outcomes between clinicians assigned to receive an active intervention involving three simulated visits with an SPI portraying a patient with acute back pain and clinicians assigned to no intervention. We opted for a passive rather than an active control condition for several reasons. First, we believe it is unlikely that the SPI visits alone without the communication content (i.e., an attention control) would affect clinician communication behaviors or subsequent test ordering. Second, we believed that implementation of an attention control would be unacceptable to clinic administration due to loss of productivity stemming from these additional scheduled visits, as SP visits use appointment slots reserved for actual patients. Third, we were concerned that clinicians randomized to the control arm would object to participating in attention control visits, which might lead to clinician attrition. After completion of data collection, we will offer clinicians in the control arm the opportunity to receive one simulated office visit with an SPI so that clinicians in the control can be instructed in the intervention communication strategies.

### Intervention description {11a}

Clinicians randomized to the control group will receive no intervention during the trial period.

Clinicians randomized to the intervention group will receive three visits over a 3- to 6-month period with an SPI scheduled during normal clinic hours for 20-min in-person office visits. (Due to the COVID-19 pandemic, clinicians may opt to have the second or third SPI visits as video visits, although we will require that the initial SPI visit be conducted in-person.) Clinicians will be aware that they are scheduled to see a standardized patient, and the SPI will begin the visit by reminding the clinician that they are an SP and orienting the clinician to the overall visit plan. During each visit, SPIs will spend 10–12 min portraying a patient with acute back pain based on pre-specified role profiles. During this time, SPIs will assess clinicians’ performance on a three-step intervention model for communicating a watchful waiting message regarding spinal imaging that is based on psychological theory [15–17], extant literature and preliminary studies [18–22], key informant interviews with clinicians, and focus groups with patients (Table 1). We consider the model to be preliminary at this stage, as we may modify content based on additional key informant interviews or our experience during SPI training and intervention pilot testing.

**Table 1:** Watchful Waiting to Avoid Inappropriate Testing (WAIT): intervention model with key skills and criteria for fulfillment

Step	Key skills	Criteria for fulfillment with examples (to guide intervention content and coding)
1. Set the stage for deferred imaging by building trust	1. Demonstrate openness and interest 2. Avoid interruptions 3. Identify the patient’s motivating concern or expectations	1. Non-verbal openness and engagement • Sits, orients toward the patient • Maintains open body position, leans in • Frequent, attentive eye contact • Engaged facial expressions or gestures (e.g., nodding) 2. Clinician does not interrupt early on. Allows patient to “tell their story” without cutting them off. 3. Clinician probes or asks for more information when patient signals a major underlying or motivating concern or expectations: “It sounds like you are worried that you seriously injured your back. Is that right?” or “You seem to be concerned that you need an MRI. Can you tell me more about that?”
2. Convey empathy	1. Legitimize patient’s concerns 2. Name and explore patient’s emotions 3. Express your understanding 4. Make supportive statements 5. Praise patient’s attempts to address pain	1. Legitimizing statements: “I can understand why you are concerned.” 2. Naming and exploring emotions: “You said you are afraid. Can you tell me more about what you are afraid of?” 3. Expressing understanding: “This is obviously a tough thing to go through. I can see that it’s really impacted your work life.” 4. Supportive statements: “I’m committed to helping you find a workable solution.” 5. Praise: “I think it’s great that you have been trying to get out and walk.”
3. Communicate optimism and openness while advocating a plan without imaging	1. Convey *optimism* when sharing your assessment and suggested plan, emphasizing reassuring aspects of the history and physical examination and the patient’s favorable prognosis. 2. Advocate a conservative treatment plan without imaging 3. If a patient asks about imaging, recommend a watchful waiting approach 4. Communicate your *availability* if the patient’s pain does not improve.	1. Frames diagnosis and treatment recommendation in an optimistic, positive frame: “Overall, I’m actually quite reassured by your history and physical. I do not see any signs of a disc problem or nerve involvement, and I’m confident that your back pain is very likely to improve markedly over the next couple of weeks.” 2. Confidently endorses an initial treatment plan that does not include imaging EX: “Given your reassuring history and exam, I’m confident that you’ll improve with conservative treatment, and in these cases, I do not recommend imaging at this time.” 3. If the patient asks about imaging, clinician advocates a “watchful waiting” approach: EX: “I do not recommend imaging at this point, but I’d consider it in a few weeks if your pain did not improve substantially, as I expect it to.” 4. Articulates a follow-up, contingency plan for what the patient should do if the pain or other symptoms worsen or do not improve. Plan should address *how* the patient should contact the clinician, *when* they should do so, and *what* the clinician is likely to do in response. (The follow-up plan may or may not include a plan for deferred imaging.) EX: “If you are pain is not substantially improved within two weeks, I’d like you to contact me via MyChart. I can then order you an x-ray and then we can have a either a phone call or a video visit.”

Titled Watchful Waiting to Avoid Inappropriate Testing (WAIT), our intervention model has three steps: (1) set the stage for deferred imaging by building trust, (2) convey empathy, and (3) communicate optimism while advocating a plan without imaging. We plan to emphasize steps 1 and 2 during the initial in-person SPI visit, which will allow the SPI to evaluate the clinicians’ use of non-verbal communication skills (step 1) and to elaborate the various ways clinicians can communicate empathy (step 2). In the second and third visits, SPIs will emphasize the skills in step 3, which involve negotiating a plan that does not include spinal imaging. To assist clinicians in grasping and remembering the skills in this step, SPIs will teach the following key words: *optimism*, *advocacy*, and *availability*. These words encapsulate the critical elements of this step, as the literature and our preliminary qualitative studies suggest that patients will accept a plan without imaging if it is confidently endorsed by the clinician, if they are provided immediate treatment options, and if the clinicians demonstrate availability and willingness to consider imaging if symptoms do not improve.

### Criteria for discontinuing or modifying allocated interventions {11b}

Clinicians will be analyzed according to their initial allocation. Modifying allocation will not be allowed.

### Strategies to improve adherence to interventions {11c}

Clinicians receiving standardized patient visits will have reduced productivity during these clinic sessions, as the standardized patient appointment will take appointment slots that could have been used by actual patients. We will therefore compensate clinicians with the equivalent of $125 in Relative Value Units (RVUs) per visit, which are used widely in the USA, to measure clinician productivity and to determine incentive compensation. We have arranged this remuneration through the participating health systems.

### Relevant concomitant care permitted or prohibited during the trial {11d}

None.

### Provisions for post-trial care {30}

None.

### Outcomes {12}

The *primary outcome* will be the rate of lumbar spinal imaging [x-ray, computed tomography (CT), or magnetic resonance imaging (MRI)] among adult patients (age ≥ 18 years) with acute back pain seen by study clinicians during an 18-month follow-up period, adjusted for the clinician’s baseline imaging rate during a 24-month pre-randomization phase. The numerator for the rates will be the number of lumbar spinal imaging tests completed, and the denominator will be the total number of patient visits with a diagnosis of acute back pain during the relevant study period. To identify this outcome, we will collect automated data from the electronic medical records of all patients seen by study physicians during the 18-month post-intervention follow-up period, as well as the 2-year period prior to the intervention (to allow for adjustment for baseline utilization). In a pilot trial [21], we abstracted testing data within 34,949 actual adult patient visits among 61 primary care physicians before and after an SPI intervention. We will pursue a similar approach here, assessing visit-level utilization of the following imaging tests: lumbar spinal MRI/CT and lumbar spinal radiography. Visits will include both in-person and telemedicine/video visits given the increased use of the latter during the COVID-19 pandemic (identified using Healthcare Common Procedure Coding System codes G2010, G2012, G2061-2063; Current Procedural Terminology codes 99421-99423, 99431, 99441-99443; 99201-99215 with a GT-95 modifier). We will collect longitudinal data on International Classification of Diseases (ICD)-9/10 diagnoses to allow identification of subsets of patients presenting with acute back pain based on the absence of back pain diagnoses on visits in the prior 6 months (ICD-9-CM: 723.1, 724.x, or ICD-10: M54.2, M54.5, M54.6, M54.89) and the inclusion of a back pain diagnostic code in the primary position on the claims, consistent with the Healthcare Effectiveness Data Information Set (HEDIS) overuse measure related to low back pain imaging [8]. We will also collect patient-level covariates to enable stratified analyses, adjustment (e.g., age, sex, available race/ethnicity, any Medicaid insurance), and restriction of analyses to patients aged 18–50 years consistent with the HEDIS overuse measure [8]. We will assess post-intervention rates of clinician ordering of both plain x-ray and advanced spinal imaging (MRI/CT).

*Secondary outcomes* will include:

1. The post-intervention rate of cervical spinal imaging among adults adjusted for baseline rate (to examine the potential for the intervention effects to generalize beyond the management of low back pain (objective 3)). We will compute this analogously to the rate of lumbar spinal imaging but only among patients with “neck pain” diagnoses in the primary position using ICD-9 and ICD-10 codes [23, 24].
2. The rate of overall diagnostic testing among adult patients adjusted for baseline. For these analyses, we will identify counts of diagnostic tests ordered during primary care visits participating clinicians during the 24-month period prior to their first SPI visits and from the date of their final SPI visits through up to 18 months, consistent with our pilot trial [21]. Specifically, we will include visit-level counts of the following diagnostic test categories: hematology and chemistry, urine and body fluid analyses, microbiology, imaging tests (subcategorized as non-spine plain x-ray or sonography, spinal x-ray, non-spine MRI or CT, neuroimaging), electrocardiography, other cardiac tests, and miscellaneous tests (e.g., nuclear medicine). We will exclude tests performed for screening or prevention (e.g., lipid or diabetes mellitus tests, bilateral screening mammograms). Both UC Davis and Sutter utilize the same EMR system (Epic), and so we expect to be able to harmonize patient and visit EMR data extracted from the two systems. The analysts compiling these data will be blinded to the study arm.
3. The post-intervention rate of test *ordering* which is distinct from test *completion*. These outcomes will be computed similarly as the test completion outcomes specified above, but we will use a signifier in the electronic medical record that a given test was ordered by the clinician. Because not all ordered tests are completed by patients, we assess test ordering as a secondary outcome.
4. Approximately 7–9 months after clinician enrolment (and after all intervention visits are completed among intervention physicians), intervention and control physicians will receive a visit by a single non-instructor SP portraying a patient with acute low back pain. Using SP observations and audio-recordings of these visits, we will ascertain as a *secondary outcome* the extent to which clinicians implemented various steps of the model. We will use iterative systematic content analysis of transcripts from audio-recorded visits to quantify how frequently clinicians engaged in the specific steps emphasized in the intervention, as well as the overall extent to which clinicians implemented watchful waiting. To guide coding, we will develop a manual to guide trained research assistants in coding transcriptions while simultaneously listening to audio-recorded visits. As the first step in the model addresses non-verbal communication, we will also incorporate standardized patient ratings on non-verbal communication. Coders will be blinded to clinician allocation. To the extent possible, we will blind SPs to the intervention group, but given the small number of total SPs, some SPs may revisit clinics where they previously performed the role of intervention SPIs, thereby making SP blinding difficult if not impossible without training and hiring entirely new SPs for the assessment phase. Inter-observer agreement for coding targeted behaviors will be assessed using Cohen’s kappa, and disagreements will be resolved by consensus or by review of the audio-recording by a third party. We have successfully used this process to assess physician-patient interaction in other studies [19, 21]. Ultimately, this process will generate a summary scale expressing the extent to which clinicians engaged in intervention steps during SP visits.
5. Six months after final SPI visits, we will survey randomized clinicians regarding the use of watchful waiting when actual back pain patients request low-value spinal imaging. We will also survey physicians regarding the use of watchful waiting for neck pain, other regional musculoskeletal pain syndromes (e.g., shoulder and knee pain), and in other contexts (e.g., when patients request antibiotics). For intervention clinicians, the survey will also inquire regarding clinicians’ views on the quality, acceptability, and utility of the SPI training. Clinician self-reported use of watchful waiting during the follow-up period will constitute a *secondary outcome* of the trial.
6. To address concerns that a watchful waiting strategy might undermine patient trust and confidence in physicians, we will assess as a *secondary outcome* for potential adverse impacts of the intervention on patient experience. To develop this measure, we will link study physicians to pre- and post-intervention patient experience data collected by the health systems as part of routine care. We have confirmed the feasibility of such linkage with UC Davis executives and are exploring this possibility within the Sutter system. These measures include visit-level Consumer Assessment of Healthcare Providers and Systems (CAHPS) results [25], which we will use to develop pre- and post-intervention summary measures of patient experience. Analyses will assess for adjusted differences in post-intervention patient experience measures among intervention physicians as compared to control physicians, after adjustment for baseline patient experience.

### Participant timeline {13}

**Fig. 1:**
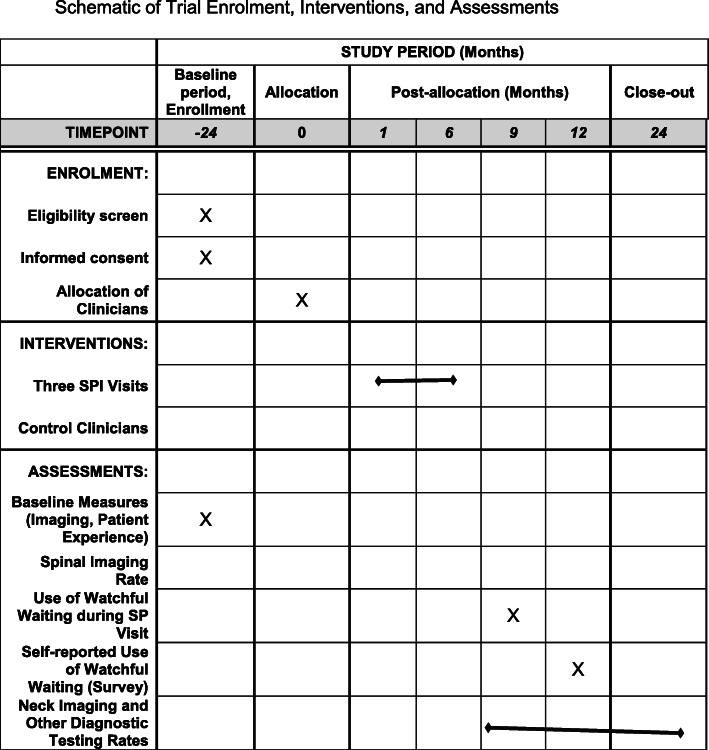
Schematic of trial enrolment, interventions, and assessments. Abbreviations: SP standardized patient; SPI standardized patient instructor

Figure 1 shows how enrolment, interventions, and study assessment will flow over an approximate period of 24 months.

### Sample size {14}

We used the exemplary dataset method in SAS to assess the power for a difference-in-difference analysis for a binary primary outcome (whether spinal imaging is ordered or not) that is assumed to have a 25% incidence in the control condition and that the effect of the intervention would be to lower the incidence by 7 percentage points. Based on empirical analysis of related data, we assumed that the outcome would have residual within-clinic and within-clinic/within-doctor correlations of 1% and 4%, respectively. We assumed hypothesis testing would be 2-tailed, with the type-1 error controlled at 5%. With 8 clinics 6 clinicians per clinic, and 92 patients per doctor (57 pre-intervention and 35 post-intervention), we would have 80.1% power to detect the effect of interest. To retain the desired sample size of 48 clinicians, we will seek to recruit at least 55 for participation.

### Recruitment {15}

We will recruit RCT clinicians during medical staff meetings (either in-person or online) and via follow-up email. During recruitment, we will inform physicians that the study will assess aspects of doctor communication but will not inform them of study hypotheses or the specific focus on spinal imaging. Similar recruitment strategies resulted in high participation rates in our previous SPI or observational studies [19, 21, 26].

## Assignment of interventions: allocation

### Sequence generation {16a}

We will stratify randomization by health system (UC Davis vs. Sutter) and primary vs. urgent care (within the Sutter system only). We will then use block randomization (in blocks of four clinicians) to ensure balance by overall, by health system, and by primary vs. urgent care.

### Concealment mechanism {16b}

Based on anticipated sample sizes, the study statistician (DJT) will use block randomization to generate three sequences of random assignments (one for UC Davis, one each for primary care and urgent care at Sutter sites). The statistician will conceal the allocations in sequentially labeled opaque envelopes and will provide these labeled envelopes to the study coordinator (CC). With each new clinician enrollment, the study coordinator will open the topmost envelope based on health system and primary vs. urgent care practice. A folded paper in the envelope will specify the clinician’s allocation to intervention or control arms.

As the study coordinator is responsible for coordinating the standardized patient interventions, neither she nor the standardized patient staff will be blinded to intervention assignments. The study coordinator will maintain a crosswalk file with clinician study identification numbers and an unlabeled binary variable indicating the study arm (coded 0 for arm #1 and 1 for arm #2). In a separate file, she will maintain a key for the binary variable indicating which codes signify the intervention and control arms, respectively. She will assign the coding to the two arms randomly by flipping a coin.

After final clinician enrollment, the study coordinator will transmit this crosswalk to the study statistician, who will use this to link the blinded study arm variable to other study datasets. The statistician and all other investigators will remain blinded to clinician allocation during initial data analysis. After completion of planned data blinded data analyses, the study coordinator will reveal to the investigators the coding for the study arm.

### Implementation {16c}

The study statistician will stratify randomization by participating health system and by primary vs. urgent care status. Within each stratum, permuted block randomization will be used.

## Assignment of interventions: blinding

### Who will be blinded {17a}

It will not be feasible to blind study clinicians to intervention and control assignments. Although we will not explicitly inform control clinicians of their assignment, these clinicians may infer their assignment as they will receive no standardized patient instructor visits.

We will blind research assistants hired to code transcripts of recorded follow-up visits with standardized patients. We will attempt to recruit standardized patients solely for the 9-month evaluation visits so that these standardized patients, who will rate clinicians on non-verbal communication, can be blinded to intervention vs. control arm. To blind coders, the project coordinator will assign a study identification number to each transcript prior to distribution to coders. The coordinator will also review the transcript for any language that may divulge the intervention assignment and will delete this from the transcript. To blind standardized patients, the project coordinator will not divulge to the standardized patients the intervention assignment of clinicians.

We will blind the study statistician to intervention assignments. The data analysts who extract diagnostic testing data from EMR databases will be blinded to intervention status. These data will be securely transferred to a project data analyst who will construct the final analytic dataset. The project data analyst will not be blinded to allocation assignments and will assign an unlabeled indicator variable to each clinic, thereby masking the intervention assignment. The final analytic dataset will be transmitted to the study statistician, and the data analyst will only divulge allocations after the study statistician has completed planned primary and secondary analyses.

### Procedure for unblinding if needed {17b}

We do not anticipate any circumstance under which the research assistants or the study statistician will need to be unblinded.

## Data collection and management

### Plans for assessment and collection of outcomes {18a}

Data for the primary outcome (spinal imaging rates) and secondary outcomes related to diagnostic testing (neck imaging, other diagnostic testing) will be derived from electronic medical record data for enrolled clinicians for the 24-month period prior to randomization and up to 18 months after the final SPI visit (or after enrollment for control clinicians). Information technology staff at the two health care systems will extract aggregate datafiles containing the required data on procedure and diagnostic codes, orders entry, and testing completion. These data will be transmitted to the study data analyst, who will develop analytic variables and a complete analytic dataset.

For the secondary outcome of clinician use of targeted communication techniques, we will use data from coded transcripts of standardized patient follow-up visits and standardized patient ratings of clinician use of non-verbal behaviors. The research assistants will be trained and supervised by a medical sociologist (M.G.). Each transcript will be coded twice by blinded research assistants with disagreement resolved by consensus or the medical sociologist. Final coding results and standardized patient ratings of non-verbal behaviors will be double entered into a Research Electronic Data Capture (REDCap) database, and at study end, these data will be transmitted to the data analyst for linkage into the analytic dataset.

Baseline and follow-up clinician surveys will be collected using REDCap surveys that clinicians will complete using an emailed weblink.

### Plans to promote participant retention and complete follow-up {18b}

Clinician retention will be encouraged by the payment of a $100 gift card and $125 of Relative Value Unit credit for each standardized patient visit as a means of compensating clinicians for lost productivity. We will offer clinicians in the control arm the opportunity to receive a single SPI visit after completion of data collection. Utilization data for clinicians who drop-out of the study will be analyzed when available, according to the assigned study arm.

### Data management {19}

Utilization data from the electronic medical record systems of the two health systems will initially be extracted in aggregate by information technology staff. These data will be transmitted to the study data analyst in an encrypted format.

The project coordinator will transmit to the data analyst double-entered and corrected datafiles containing data from standardized patient follow-up encounters. The data analyst will have access to the REDCap database containing clinician survey responses.

With the direction of the principal investigator and the study statistician, the data analyst will develop analytic variables, link the datasets, and develop a final, blinded analytic dataset. The analyst will transmit this dataset to the study statistician using encrypted file transfer protocols.

All datasets will be stored on password-protected computers behind University-based firewalls.

### Confidentiality {27}

The project coordinator will maintain data on potential and enrolled clinicians in password-protected computers in research offices at the UCD Medical Center. Similar files will be maintained by the study coordinator at the Sutter Institute for Medical Research. Both institutions maintain the highest level of cybersecurity with computers protected behind certified firewalls. All participants will be identified by unique study identification numbers that will be included in a password-protected file maintained by the study coordinators at each site. All other research datafiles will contain only the study identification number, reducing the risk of breach of confidentiality of individual participating clinicians. Research coordinators will access the identifying information only for essential trial activities, such as communication with participating clinicians about upcoming standardized patient visits, compensating clinicians for study visits, collection of pre- and post-trial surveys, and study closure.

### Plans for collection, laboratory evaluation, and storage of biological specimens for genetic or molecular analysis in this trial/future use {33}

Not applicable.

## Statistical methods

### Statistical methods for primary and secondary outcomes {20a}

Analyses will be conducted using the intention-to-treat principle that participants are considered to belong to the group to which they were assigned. For the primary outcome (imaging rates among randomized clinicians), the unit of analyses will be primary care visits among actual patients seen during the post-intervention period (nested within clinicians and clinics). The primary outcome will be within-visit binary indicator of whether any spinal imaging was ordered and completed (i.e., plain lumbar spinal x-rays, spinal MRI or CT) among patients with acute low back pain during the post-intervention period. We will similarly obtain baseline data for patients seen by randomized clinicians during a 2-year pre-intervention period. Using a Generalized Linear Mixed Model (GLMM) with a Poisson distribution and log link along with random effects for units of randomization and, possibly, for clinicians nested within units of randomization, we will test for intervention effects by testing for the significance of an interaction term between a categorical variable for the intervention group and a covariate distinguishing pre- and post-intervention periods. We will examine these outcomes among all adult patients in the practice with visits with acute back pain diagnoses as well as the subset of adult patients aged 18–50 years (consistent with the HEDIS overuse measure) [8]. We will take a similar approach to analyzing secondary outcomes of post-intervention use of advanced neck imaging and any diagnostic testing. Insofar as they enhance precision in estimation of intervention effects, we will also include as fixed-effects in each model baseline clinician characteristics. We will assess regression-adjusted intervention main effects (vs. control) using a two-sided hypothesis test at *α* = 0.05 and estimated with 95% confidence intervals. We will not adjust for multiple comparisons in this developmental study but will present all quantitative results in a single publication to facilitate judicious interpretation.

Secondary outcomes include (1) clinician implementation of specific watchful waiting techniques based on coded audiotapes and standardized patient ratings (likely a summary scale), (2) clinician self-report of the use of watchful waiting with actual patients (an ordinal measure), and (3) actual patient satisfaction with their recent experience with clinicians (likely a *z*-score based on seven survey items). When there is a single secondary outcome per clinician, we will analyze for intervention effects using ordinal or logistic regression as indicated by the response variable within a GLMM model with random intercepts for the units of randomization. For analyses of patient experience, we will also use generalized linear mixed models (GLMM) with similar random effects specified as above and with the distribution and link functions tailored to the outcome variable (Gaussian for the *z*-score outcome) [27, 28].

### Interim analyses {21b}

No interim analyses will be performed.

### Methods for additional analyses (e.g., subgroup analyses) {20b}

Although our sample of urgent care clinics will be small (likely two), we will plan to conduct analyses of the primary outcome with clinicians stratified as primary care vs. urgent care. We will also conduct a subgroup analyses with clinicians stratified by years of experience (e.g., above or below the median years of experience for the clinician sample) and physicians vs. nurse practitioners/physician assistants. If many intervention visits are delivered by video, we will assess whether the modality of intervention delivery (in-person vs. video) modified intervention efficacy.

### Methods in analysis to handle protocol non-adherence and any statistical methods to handle missing data {20c}

In case of significant protocol non-adherence, secondary analysis will be conducted to estimate “per protocol,” “as-treated,” or adherence-weighted intervention effects. Our primary analysis will analyze all available data, assuming missingness at random conditional on observed data. If item-level missingness exceeds 5% for an outcome, we will conduct multiple imputation and use sensitivity analysis to estimate effects under a range of alternative assumptions about the missingness model [29].

### Plans to give access to the full protocol, participant-level data, and statistical code {31c}

We will be willing to share de-identified participant-level data and statistical code after publication of initial trial results.

## Oversight and monitoring

### Composition of the coordinating center and trial steering committee {5d}

This two-site trial will be coordinated at the Center for Healthcare and Policy Research at the University of California (UC), Davis Medical Center. Recruitment and data collection at the Sutter site will be coordinated by the Sutter Institute for Medical Research. UC Davis staff will take primary responsibility for study supervision, coordination, and data management. There is no trial steering committee or endpoint adjudication committee.

### Composition of the data monitoring committee, its role, and reporting structure {21a}

Our intervention addresses clinical communication behavior and ordering of non-recommended diagnostic tests. As such, the IRB judged trial participation to pose minimal risk and did not require a data safety monitoring board.

### Adverse event reporting and harms {22}

During informed consent, participants will be provided the contact number for the study coordinators at each site and the principal investigator. If any adverse events or unintended consequences are reported, the principal investigator will immediately consult IRB officials about appropriate responses. In light of the COVID-19 pandemic, we plan to train all standardized patients in symptoms and signs of COVID-19, social distancing protocols, usage of face coverings, and the omission of physical examination during visits. Nevertheless, the possibility exists that a standardized patient may expose a participating clinician to the SARS-Cov2 virus. We anticipate that the risks to clinicians will be minimal, as standardized patients will use COVID-19 preventive precautions. Moreover, medical staff at both sites routinely wear masks and face shields during in-person visits. Nevertheless, in the event of clinician exposure to a standardized patient with COVID-19, the principal investigator will immediately notify the IRB, infection control officials at UC Davis, and will contact the study clinician with specific recommendations about recommended monitoring or potential testing.

### Frequency and plans for auditing trial conduct {23}

Trial conduct will not be audited independently. The investigators will submit yearly progress and financial supports to the sponsor (the Agency for Healthcare Quality and Research).

### Plans for communicating important protocol amendments to relevant parties (e.g., trial participants, ethical committees) {25}

The investigators would submit any protocol changes or amendments to the IRB for approval. They would also update the ClinicalTrials.gov protocol promptly.

## Dissemination plans {31a}

The investigators plan to disseminate the trial results by publication in the peer-reviewed literature. Dissemination of results to clinicians and policymakers may also be promoted by issuance of a press release at the time of publication along with outreach via social media outlets. The investigators also intend to present the study results at national research meetings of primary care and health services researchers. The results will also be included in the ClinicalTrials.gov database. There are no publication restrictions affecting the trial.

## Discussion

This trial will test the effect of an educational intervention designed to improve clinician skill in counseling patients with acute back pain in a manner that meets patients’ informational and emotional needs without ordering inappropriate spinal imaging. The intervention will consist of three simulated visits with a standardized patient instructor who will evaluate clinician performance on targeted skills and provide responsive feedback. We have nearly completed the qualitative studies which have assisted in the identification of the targeted skills and will soon begin recruitment of clinicians and the hiring and training of the standardized patients. We plan to recruit 55 clinicians within 8–10 clinics within two health systems in the Sacramento region. The clinicians will be randomized to intervention and control arms.

The success of the intervention may hinge on the quality and skill of the standardized patient instructors. The instructors not only have to portray patients with back pain accurately but must simultaneously assess clinicians’ fulfillment of targeted skills, ranging from non-verbal communication to the delivery of optimistic messaging when discussing back pain prognosis. The instructors must also teach the doctors the key steps in the model and provide formative feedback. Appreciating the difficulty of these tasks, we have hired a standardized patient trainer who has experience in intervention studies involving SPIs. We have also developed a training manual for use by instructors during the training period. We anticipate that training each SPI will require multiple mock encounters with clinician investigators or volunteer clinicians. The standardized patient trainer will also closely monitor audio-recordings of early study visits to ensure role fidelity and the delivery of appropriate feedback to clinicians based on skill performance.

The primary outcome of the trial is the spinal imaging rate among actual back pain seen by study clinics after the intervention. To identify this outcome, we will abstract test ordering and completion data from the electronic medical records of two health systems. Secondary utilization outcomes will also derive from electronic medical record data. While each system uses the same electronic record, we anticipate some logistical challenges in ensuring abstraction of equivalent data in each system. We are developing relationships with information technology leaders at each site, and as data extraction begins, we will hold regular meetings with information technology staff at the two sites to engender a team approach to the data extraction. Analysts at the two sites will be encouraged to communicate regularly outside of team meetings and to work simultaneously and collaboratively in the extraction of required data elements.

The COVID-19 pandemic poses some operational challenges. While we anticipate that clinicians will be willing to participate and to receive SPI visits in-person, some clinicians may opt to have some of the SPI visits via video technology. We have explored the feasibility of implementing video visit technology at each site and do not anticipate difficulties. However, we have some concerns that clinicians may be less responsive to instructor feedback if delivered via video as compared to in-person. We will therefore require that at least the initial SPI visit be conducted in-person, and if many SPI visits are delivered using video technology, we will conduct secondary subgroup analyses to assess whether modality modifies intervention efficacy. The pandemic will also necessitate additional training of SPs in hygiene and social distancing precautions to minimize SPI and clinician infection risk during SP visits.

If the intervention reduces the rate of spinal imaging among actual back pain patients (the primary outcome), then SP-based training within simulated office visits may be a feasible means of reducing the overuse of this diagnostic test in clinical practice. Compared to other quality improvement interventions, the intervention is efficient with clinician time (60 min over 3 visits), and clinicians have been receptive to SP interventions during routine office hours, as they require no out-of-office time commitment. Clinicians receiving prior SPI interventions have also found to be fun, instructive, and relevant to practice [21, 26].

Our analysis plan will assess whether the intervention affects other test ordering, potentially because clinicians generalize lessons learned in counseling acute back pain patients to other patients with complaints that prompt consideration of low-value tests (e.g., neck pain). A finding that the intervention affects both the primary and secondary outcomes would be particularly significant, as the attractiveness of the intervention to health systems and policymakers would be much greater if intervention effects are not isolated to low back pain imaging. If our trial finds significant impacts on both primary and secondary utilization outcomes, then results would justify confirmatory trials and dissemination attempts with non-randomized evaluations.

## Trial status

This protocol is dated October 9, 2020. Recruitment of clinicians and clinics began on November 4, 2020, and is anticipated to continue through May 31, 2021.

## Abbreviations

CAHPS: Consumer Assessment of Healthcare Providers and Systems
CT: Computed tomography
FTE: Full-time equivalent
ICD: International Classifications of Diseases
IRB: Institutional Review Board
MRI: Magnetic resonance imaging
RCT: Randomized controlled trial
SP: Standardized patient
SPI: Standardized patient instructor
UCD: University of California, Davis
